# Expression of calcium binding protein S100 A7 (psoriasin) in laryngeal carcinoma

**DOI:** 10.1590/S1808-86942012000400012

**Published:** 2015-10-20

**Authors:** Rogério Costa Tiveron, Luiz Carlos Conti de Freitas, David L Figueiredo, Luciano N Serafini, Rui Celso Martins Mamede, Marco A Zago

**Affiliations:** MD, PhD (Department of Ophthalmology, Otorhinolaryngology and Head and Neck Surgery - School of Medicine of Ribeirão Preto of University of São Paulo - Brazil); MD, PhD (Department of Patology - School of Medicine of Ribeirão Preto of University of São Paulo - Brazil); MD, PhD (Department of Internal medicine - School of Medicine of Ribeirão Preto of University of São Paulo - Brazil)

**Keywords:** laryngeal neoplasms, carcinoma, S100 proteins

## Abstract

Many studies have reported increased expression of S100 A7 (psoriasin) in neoplastic lesions. Among them are studies on breast carcinoma, bladder squamous cell carcinoma, skin tumors and oral cavity squamous cell carcinoma. The expression of S100 A7 has not been described for laryngeal cancer.

**Objective**: This study aims to identify the expression of the calcium-binding protein S100 A7 and its correlation with squamous cell carcinomas of the larynx.

**Material and Methods**: Specimens from 63 patients were submitted to immunohistochemistry testing with antibody S100 A7. Results were classified and compared.

**Results**: The group with highly differentiated tumors had the highest treatment failure scores. Moderately differentiated tumors had higher treatment failure scores than poorly differentiated tumors. Higher scores were predominantly seen on stages I and II in moderately differentiated tumors, whereas score distribution was more homogeneous in advanced stage disease (III and IV). Regarding failure in treatment, the group scoring zero (3/4 complications: 75%) differed significantly from the remaining groups (13/59: 22%).

**Conclusions**: S100 A7 marker was expressed in 93.7% of laryngeal cancer cases, with higher positive correlation rates in more differentiated tumors and significantly lower rates of treatment failure. Scores had no impact on survival rates.

## INTRODUCTION

Despite the multiple advances in the diagnosis and treatment of laryngeal tumors, the survival rate of affected patients has increased little over the last 30 years. This is believed to be due to the heterogeneous course of the disease, and it is of fundamental importance to identify the most aggressive ones in order to improve their cure rate by applying a specific treatment. Many clinical and histopathological characteristics that might define this group of tumors have been intensely studied, with emphasis on cellular and molecular alterations over the last few years, although without success^1^.

The study of the psoriasin protein is still in an early stage^2^; however, its presence in urine suggests that it is a noninvasive marker capable of identifying cases of cancer and, according to Skliris et al.^3^, by being regulated by the activity of the estrogen receptor, this gene may represent a guide for target therapy. Frequent reports of increased expression of this gene in neoplastic lesions have been published. Among them are studies of ductal carcinoma of the breast, spinocellular carcinoma of the bladder, skin tumors and spinocellular carcinoma of the oral cavity^4^, ^5^.

On this basis, the objective of the present study was to identify the immunohistochemical expression of the psoriasin S100 A7 in spinocellular carcinomas of the larynx (SCCL) and its correlation with demographic variables of the patients and with the histological characteristics of the tumor. An additional objective was to compare the overall survival curves of patients who express this gene to those of patients who do not express it.

## MATERIALS AND METHODS

We selected 63 patients with a histopathological diagnosis of SCCL surgically treated who gave written informed consent to participate in the study (Protocol CEP n^o^ 9371/2003). Inclusion criteria were the availability of a representative tissue sample for histological processing, a precise definition of the primary tumor site, and complete clinical and follow-up data. The specimens were routinely processed for anatomopathological study. The clinical, sociodemographic, anatomopathological and follow-up data of these patients were stored in a data control system.

For the immunohistochemical reactions, serial 4 mm sections were obtained from the representative block and mounted on slides with an adhesive 3-amino-propyltriethoxy-silane solution (Sigma Chemical Co.®, St. Louis, MO). The sections were submitted to antigen retrieval with heat for 3 minutes in a steamer using a 10 mM citric acid solution, pH 6.0. Endogenous peroxidase was blocked with a 6% aqueous solution of hydrogen peroxide. The antibody used for the immunohistochemical reaction was S 100 A7 (psoriasin) - liquid mouse monoclonal antibody NCL-L-S100 A7 (Novocastra Laboratories, Newcastle-upon-Tyne, UK). A dextran polymer and the NovoLink enzyme amplification system (Novocastra) were used to prevent an endogenous reaction due to the presence of biotin and avidin. The material was incubated with the primary antibody at 37°C in a moist chamber overnight and with a secondary antibody diluted in PBS for 30 minutes at 37°C. After washing with PBS, the slides were incubated with the Novolink amplifier system at 37°C for 30 minutes. Finally, the sections were washed in running water for 10 minutes, counterstained with Harris hematoxylin for 10 seconds, washed again in running water, dehydrated with ethanol, cleared with xylene, and mounted with Permount resin (code S15-100, Fisher Scientific®, Fair Lawn, NJ). Positive controls (normal skin) were used for each reaction batch. Negative controls (blanks) were prepared by replacing the primary antibody with PBS.

**Figure 1:**
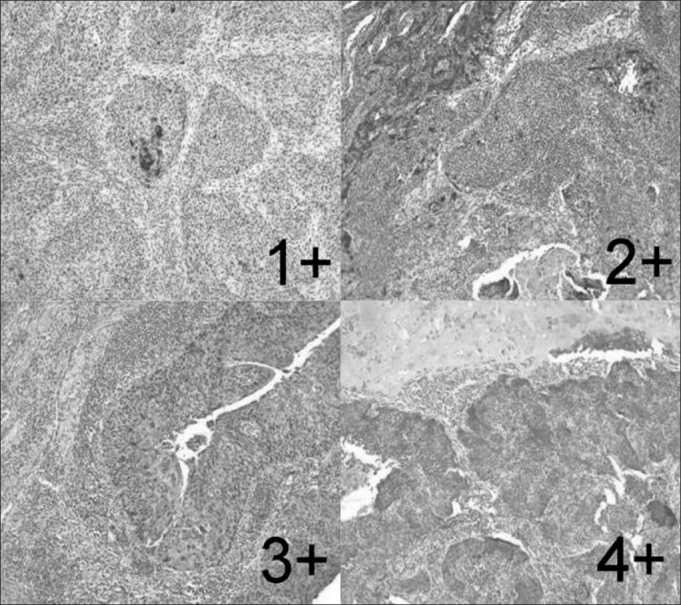
Larynx's specimen of spinocellular carcinoma after immunohystochemical reaction with expression of psoriasin S100 A7. Escores 1+, 2 +, 3+ and 4+ (Zoom 100X).

The reactions were examined by two pathologists who were unaware of the cases and defined by consensus. The reaction were scored subjectively as zero (up to 5% of the neoplastic cells labeled), 1+ (5-25%), 2+ (25-50%), 3+ (50-75%), and 4+ (more than 75%) (Figure 1). A positive immunolabeling above 5% was considered for statistical analysis.

The different reaction scores obtained were compared to the clinical, histopathological and follow-up data of the patients. Data were analyzed statistically by nonparametric methods since the score variable did not show a normal distribution. The score variable was analyzed as a numerical variable. The level of significance was set at *p* ≤ .05.

## RESULTS

### Characteristics of the Sample

The sample consisted of 63 patients, 58 men aged 27 to 78 years (mean; 57.46 years) and five women aged 47 to 59 years (mean; 53.2 years). Most patients had tumors located in the supraglottis (36 case) and in the glottis (24 cases), with a respective incidence of 57 and 38%. Only three cases (4.7%) had tumors originating in the subglottis. Regarding clinical stage, eight cases were stage I, eight were stage II, 22 were stage III, and 25 were stage IV, three of them being IVb. The glottic tumors were distributed among all stages, with eight of them being classified as stage I; among the supraglottic tumors, 15 were stage III and 15 were stage IV. Only two of the 63 patients were non-smokers and 52 were alcohol drinkers.

Of the 63 patients studied, 17 (27%) died, 11 of them (17.4%) due to the neoplasia and six (9.5%) due to causes not related to the neoplasia. Local recurrence occurred in 10 cases, seven patients had regional recurrence and one had distant metastases. A second primary tumor occurred in five cases.

All tumors were classified histologically according to degree of differentiation, with 13 (20.6%) being well differentiated (WD), 37 (58.7%) moderately differentiated (MD), and 13 (20.6%) poorly differentiated (PD). Most of the glottic tumors (91.7%) were moderately differentiated (16/24 = 66.7%) or well differentiated (6/24 = 25%) and only two were poorly differentiated. The supraglottic tumors were more uniformly differentiated, with seven (19.4%) being well differentiated, 19 (52.7%) moderately differentiated and 10 (27.8%) poorly differentiated. Among the subglottic tumors, two were moderately differentiated and one was poorly differentiated.

### Score Determination

The score was 0+ for four cases (6.3%), 1 + for 10 cases (15.9%), 2+ for 15 cases (23.8%), 3+ for 10 cases (15.9%), and 4+ for 24 cases (38.1%). The scores obtained (Table 1) were related to the clinical and sociodemographic variables of the patients and to the histopathological characteristics of the tumors. The distribution of the scores was quite similar for the various anatomical sites, except for the subglottis due to the scarce number of cases. Glottic tumors tended to be classified as 3+ and 4+ (15 of 24), whereas the supraglottic tumors tended to have a low score (20 of 36).

**Table 1:** Score distribution, degree of differentiation, tumor location, stage, failure in treatment and follow up of studied patients.

Patient	Score	Degree of Differentiation	Tumor location	Stage	Failure+ Complication	Follow up
1	4+	WD	Supraglottic	III		DFS
2	2+	MD	Supraglottic	IVa	LR + SPC	Death from cancer
3	4+	MD	Subglottic	IVa		DFS
4	4+	MD	Supraglottic	III	LR	Death from cancer
5	4+	MD	Glottic	IVa		DFS
6	4+	MD	Glottic	IVa	LR + NR	Death from cancer
7	1+	PD	Supraglottic	III		DFS
8	4+	MD	Glottic	I		DFS
9	2+	MD	Supraglottic	III		DFS
10	4+	MD	Supraglottic	II		DFS
11	1+	PD	Supraglottic	II		DFS
12	2+	MD	Supraglottic	IVa	Death from surgery	
13	3+	MD	Glottic	IVa	SPC	DFS
14	3+	MD	Supraglottic	II	LR + NR	Death from cancer
15	Zero	PD	Supraglottic	III		DFS
16	1+	PD	Supraglottic	II		DFS
17	Zero	PD	Glottic	III	LR + NR	Death from cancer
18	4+	MD	Supraglottic	IVa		DFS
19	4+	MD	Supraglottic	IVb		DFS
20	3+	MD	Glottic	II		DFS
21	2+	MD	Glottic	I		Death from other causes
22	1+	MD	Supraglottic	III		Death from other causes
23	1+	PD	Supraglottic	II		DFS
24	4+	WD	Supraglottic	III	Distant Metastases	Death from cancer
25	2 +	PD	Supraglottic	IVa	Death from surgery	
26	1+	PD	Supraglottic	IVa	SPC	DFS
27	3+	MD	Glottic	I		DFS
28	4+	WD	Glottic	I		DFS
29	3+	MD	Subglottic	IVa		DFS
30	Zero	PD	Supraglottic	III	LR + NR	DFS
31	2+	MD	Supraglottic	III		DFS
32	1+	MD	Supraglottic	IVa		DFS
33	4+	WD	Glottic	I		DFS
34	1+	PD	Supraglottic	IVa	LR	Death from cancer
35	2+	PD	Supraglottic	IVa		DFS
36	2+	MD	Supraglottic	III		DFS
37	2+	MD	Glottic	I	SPC	Death from cancer
38	2+	MD	Glottic	III	LR + NR	DFS
39	3+	WD	Subglottic	III		Death from other causes
40	4+	WD	Supraglot	II		DFS
41	4+	WD	Glottic	III		Death from other causes
42	4+	MD	Glottic	I		DFS
43	4+	WD	Glottic	IVa		DFS
44	4+	WD	Supraglottic	IVa	Death from surgery	
45	4+	WD	Supraglottic	IVa		DFS
46	4+	WD	Supraglottic	III		Death from other causes
47	4+	MD	Supraglottic	IVa	NR	Death from cancer
48	1+	PD	Glottic	IVa		DFS
49	2+	MD	Glottic	III		DFS
50	2+	MD	Glottic	IVa		DFS
51	4+	WD	Glottic	I	LR	DFS
52	3+	MD	Glottic	III		DFS
53	3+	MD	Supraglottic	III		DFS
54	Zero	MD	Glottic	IVb	LR + NR	DFS
55	4+	MD	Supraglottic	IVa		DFS
56	3+	MD	Supraglottic	III		DFS
57	4+	WD	Glottic	II		DFS
58	2+	PD	Supraglottic	III		DFS
59	4+	MD	Glottic	IVa		DFS
60	2+	MD	Supraglottic	IVa	SPC	DFS
61	3+	MD	Supraglottic	III		DFS
62	1+	MD	Glottic	III		DFS
63	2 +	MD	Supraglottic	IVa		DFS

LR: Local Recurrence; NR: Neck Recurrence; SPC: Second Primary Cancer; DFS: Disease Free Survival; WD: Well differentiated; MD: Moderately Differentiated; PD: Poorly Differentiated.

The score was compared to the degree of cell differentiation by the nonparametric Kruskal-Wallis test, whose results are presented in Table 2. There was a statistically significant difference between groups. The score for the WD group was significantly higher than the score for the MD and PD groups and the MD group had significantly higher scores than the PD group (WD > MD > PD). Poorly differentiated tumors were in the low score range, i.e., none of them had a 3 or 4+ score. Considering only moderately differentiated tumors, analysis of the score in relation to staging is presented in Table 3, where stages I and II, stages III and IV and scores 0, 1 and 2+ and 3 and 4+ are grouped.

**Table 2:** Distribution of the scores according the degree of cell differentiation by the nonparametric Kruskal-Wallis test.

Degree of Differentiation	N	Median	Maximum	Minimum	Mean	SD
PD	13	1.00	2.00	0.00	1.00	0.71
MD	37	3.00	4.00	0.00	2.76	1.09
WD	13	4.00	4.00	3.00	3.92	0.28

$\chi^{2}$ = 32.90, *p* < .001*, N: Number of individuals; SD: Standard deviation; PD: Poorly differentiated; MD: Moderately differentiated; WD: Well differentiated.

**Table 3:** Score distribution according to the tumor stages for MD Tumors.

Staging*	Score 0/1+/2 +	Score 3+/4+
I/II	2	6
III/IV (IVa and IVb)	14	14

MD: Moderately differentiated;

* TNM (American Joint Committee on Cancer).

No significant relations were detected between the scores and the following variables: tumor site, clinical stage, patient age, presence of regional and/or local recurrence, second primary tumor, and death. Table 4 shows the correlations between failure in treatment such as the presence of local or regional recurrence, metastases and a second primary tumor and the different scores. There was a statistically significant difference (*p* = .02) between a zero score (3/4 = 75%) and the remaining ones (15/59 = 25.4%) and failure in treatment.

**Table 4:** Score distribution according to presence of failure in treatment (local or regional recurrence, metastases and second primary tumor).

Score	Number of Patients	Failure in Treatment
Zero	4	3
1+	10	2
2+	15	6
3+	10	2
4+	24	5

The various curves were compared by the Kaplan-Meier method and the log rank test. No significant difference in the survival was observed when the scores were analyzed separately or as a whole. Two groups were considered for the comparison of the survival curves obtained between score groups: group 1 with absent or low positivity (considering zero, 1+ and 2+ scores) and group 2 with strong positivity (considering 3+ and 4+ scores).

## DISCUSSION

Psoriasin S100 A7 expression was observed in 93.7% of the samples analyzed and its scores were distributed among the various anatomical sites, except the subglottis due to the small number of cases. Glottic tumors showed a greater tendency to be classified as 3 and 4+ (15/24 = 62.5%), whereas supraglottic tumors tended to show lower immunolabeling scores (20/36 = 55.5%). Poorly differentiated tumors received lower scores. This explains the greater tendency of glottis tumors to receive a higher score since more of them were well differentiated. Similarly, supraglottic tumors had lower scores since more of them were poorly differentiated, although no significant difference in score was observed in relation to tumor site. This fact is explained by the probable function of this gene, which acts while the cells are well differentiated. As stated by Moubayed et al.^4^, this gene does not seem to have a function in undifferentiated tumors.

Of the 37 moderately differentiated tumors, 19 (19/36 = 53.7%) were located in the supraglottis, 16 (16/24 = 66.6%) in the glottis and 2 in the subglottis. Most of these tumors (56.7%, 21/37) were classified as more than 2+; however, analysis of their distribution according to clinical stage revealed that those classified as stage I or II tended to obtain a score of more than 2+ (6/8), while those classified as stages III and IV showed a more uniform score distribution. In other words, the scores decreased with increasing tumor mass. Thus, the action of the psoriasin gene seems to be concentrated in the early phases of carcinogenesis.

Rigopoulou et al.^6^ detected susceptibility factors for papillary thyroid carcinoma that varied according to the populations investigated (Spaniards, Americans and Germans), showing that this susceptibility depended on genetic and environmental factors. This analysis was not possible in the present study because only two patients were non-smokers. No effect of alcohol drinking on psoriasin gene expression was detected here among alcoholic patients. Also, no significant relation was observed between patient age and tumor score.

Although the clinical follow-up of the present patients was shorter than that recommended in the literature, comparison of survival rates according to the scores obtained (analyzed individually or in groups) did not demonstrate a statistically significant difference.

### Failure in Treatment

The survival of the present sample was 73% (46/63) and the death rate was 27% (17/63 = 27%). Death was due to failure in treatment in 12.7% (8/63), complications related to treatment in 4.7% (3/63), and to other causes in 9.5% (6/63). Thus, 64.7% (11/17) of the deaths were due to the tumor or to its treatment.

Of the 63 patients studied, 25.3% (16/63) had some type of failure intreatment (10 local recurrences, 7 cervical recurrences, 1 metastasis, and 5 second primary tumors, for a total of 23 events in 16 patients) and 7 are still alive. Among the cases of failure in treatment, local or regional recurrence (from now on simply referred to as “recurrence”) was observed in 10 cases, while distant metastases and the occurrence of a second primary tumor (from now on referred to as “others”) was observed in 5, with one patient had simultaneous recurrence and a second primary tumor. Distribution of those events was similar among the glottis (5 recurrences and 2 others) and the supraglottic (6 recurrences and 4 others). There were not observed in the subglottis.

Considering only the presence of failure intreatment (detected in 16 cases), it can be seen that treatment failure occurred in 29.1% (7/24) of the glottic tumors and in 25% (9/36) of the supraglottic tumors. An explanation for the greater aggressiveness observed among the glottic tumors is that these tumors are not treated with radiotherapy or endoscopic surgery (a treatment indicated for earlier stages). On this basis, the sample of glottic tumors studied here probably consisted of the more aggressive fraction of these tumors.

No difference was observed between alcohol drinkers and non-drinkers regarding the various factors studied. Thus, even though alcohol has a proven role in the etiology of cancer of the larynx, it had no impact on the survival of the present patients, in contrast to the opinion of Dikshit et al.^7^.

Treatment failure occurred in 25% (2/8) of stage I tumors, in 12.5% (1/8) of stage II tumors, in 22.7% (5/22) of stage III rumors, and in 28% (7/25) of stage IV tumors. The failure observed in stage I concerned the glottic tumors, when an attempt was made to treat them by partial surgery of the larynx. It is known that supraglottic carcinomas have a different biological behavior compared to carcinomas of the larynx, always showing greater aggressiveness due to the frequent metastatization to lymph nodes, as reported by Greenman et al.^8^. However, other mechanisms are believed to be involved in this aggressiveness, such as a higher incidence of stage III and IV tumors compared to glottic tumors, since supraglottic tumors are diagnosed later when the volume of neoplastic cells is greater. In the present study, most supraglottic tumors were in stages III and IV, but did not show greater aggressiveness.

The scores in the group who had failure in treatment were zero+ in 75% of cases (3/4), 1+ in 20% (2/10), 2+ in 26.6% (4/15), 3+ in 20% (2/10), and 4+ in 20.1% (5/24). These numbers lead us to conclude that failure in treatment were significantly more frequent among patients with a zero score for the psoriasis gene. It should be emphasized that local recurrence is a consequence of the surgical act when scarce safety margins are left in the surgical bed, thus being related much more to the indication and execution of surgery than to tumor aggressiveness. This is not the case for regional recurrence which, although related to the indication and execution of cervical lymph node dissection, is due to greater tumor aggressiveness. Other failure associated to treatment in which erroneous indication of treatment is not involved are distance metastatization and the presence of a second primary tumor. These three complications, i.e., regional recurrence, metastasis and second primary tumor, were present in 13 cases, i.e., in 19.4% of supraglottic tumors (7/36) and in 25% (6/24) of glottic tumors. They affected 8 cases with a score of less than 3+ (8/29 = 27.6%) and 5 cases with a score of more than 2+ (5/34 = 14,7%). The occurrence of statistically significant complications was confirmed in cases with a zero+ psoriasin score and the complications also tended to occur among patients with a score < 3+.

Of the tumors analyzed, 20.6% were well differentiated, 58.7% were moderately differentiated and 20.6% were poorly differentiated. The distribution of cell differentiation was closely similar in the population of patients who presented failure in treatment, with the tumors being well differentiated in 23% of them (3/13), moderately differentiated in 27% (10/37), and poorly differentiated in 30% (4/13). It shows that cell differentiation had no influence on failure in treatment. The distribution of cell differentiation was the same in each anatomical region. There is no evidence that intrinsic biological differences exist in tumors from these different sites. However supraglottic tumors tend to be more undifferentiated than glottic tumors, a fact that was not observed in the present study.

### Psoriasin

In the present study, the expression of the psoriasin marker (S100 A7) was detected in 93.7% of SCCL cases, being strong in differentiated tumors and weak in poorly differentiated ones. The more intense expression of psoriasin in differentiated tumors agrees with the results obtained by Leygue et al.^9^ who detected a strongly positive expression of psoriasin in ***in situ*** carcinomas and a lower expression in invasive carcinomas. These authors evaluated the expression of psoriasin mRNA in frozen breast tissue biopsies from ***in situ*** and invasive ductal carcinomas and from normal tissues. More differentiated tumors are known to tend to be less aggressive, with a better patient course and survival. However, this fact was not confirmed in the present study since we did not detect differences in survival between the groups studied. Watson et al.^10^ reported that the expression of psoriasin was extensively detected in ***in situ*** tumors but was only expressed in 18% of invasive tumors and suggested that this expression is associated with changes in epithelium differentiation and must play a role in early tumor progression. An additional factor for its involvement in the progression of ductal carcinoma is its location in a region of chromosome 1 that frequently (> 50% of cases) presents loss of heterozygosity in invasive tumors^11^.

In a recent study, Moubayed et al.^4^ determined the expression of psoriasin mRNA by RT-PCR in skin biopsies from patients with preneoplastic lesions, with spinocellular carcinomas and basocellular carcinomas and in normal controls. They detected increased expression in patients with preneoplastic lesions and with basal cell carcinoma and a significantly increased expression in spinocellular carcinoma. The authors concluded that, as is the case for ductal breast carcinomas and spinocellular carcinomas of the bladder, the increase in the expression of psoriasin must play an important role in the progression of skin cancer. Watson et al.^10^ also believe that psoriasin has an important effect on the development of the invasive phenotype, which may be both an indirect effect on the immune cell response of the host or even a direct effect contributing to tumor invasion. In this sample, among moderately differentiated tumors, expression tended to decrease with increasing tumor stage. The observation that 75% of the tumors with a zero score had failure in treatment shows that tumoral invasion is favored by the absence of psoriasin.

According to Zhou et al.^1^, ^2^, ^3^, ^4^, ^5^, ^6^, ^7^, ^8^, ^9^, ^10^, ^11^ calcium-binding protein acts on transduction and thus on the carcinogenesis of the epithelium, but its function has not been fully determined. According to Petersson et al.^12^ expressed psoriasin develops a phenotype of resistance to apoptosis and, according to Wolk et al.^13^ the lack of expression inhibits cell differentitiation and increases cell motility, facilitating invasion. Thus, psoriasin is highly expressed in differentiated tumors during the pre-invasive phase and less expressed in less differentiated tumors and tumors in the invasive stage^4^. Krop et al.^14^ believe that psoriasin acts by promoting angiogenesis and by detaching cells that govern the anti-invasive function, thus explaining the high expression of the gene in well differentiated or estrogen-negative tumors.

According to Fukuzawa et al.^15^, the psoriasin gene acts on transcription and the segment of the gene that exerts this function in spinocellular carcinomas of the oral cavity is -1513 to 988. According to Mandal et al.^16^, psoriasin modulates the immune response favoring the growth of breast cancer in the initial phases. However, Zhou et al.^3^ demonstrated that psoriasin acts by inhibiting, or being inhibited by, the beta catenin complex. Thus, according to these authors, in situations of high psoriasin expression there is a reduction of the beta catenin complex and the preneoplastic tissue is transformed into a well differentiated spinocellular carcinoma of little aggressiveness. A spinocellular carcinoma will be poorly differentiated and highly aggressive when the expression of the beta catenin complex is so high as to inhibit the expression of psoriasin.

In our opinion, this gene acts on the initial phases of tumorigenesis, being important in the origin of differentiated tumors. However, as the tumors become undifferentiated, the influence of the gene disappears or the genes are consumed for the process to occur. This must have been the mechanism of action of the psoriasin gene involved in laryngeal tumors, i.e., the gene participates in the differentiation of the tumors and the tumors become more aggressive as the expression of the gene is reduced. The present study was the first to evaluate the expression of this marker in a laryngeal neoplasia and further studies may obtain complementary information that will make this gene, expressed in 93.7% of the present cases, a marker of the presence and aggressiveness of SCCL.

## CONCLUSIONS

The psoriasin S100 A7 marker was expressed in 93.7% of the present SCCL cases, with a higher positivity in well differentiated tumors, tending to decrease with increasing stage in moderately differentiated tumors. Failure in treatment was more frequent among patients with a zero score, although the score had no impact on survival.
